# PD57 Drugs For Chronic Degenerative And Rare Diseases In The Brazilian Public Health System: Profile Of Budget Impact Analyses

**DOI:** 10.1017/S026646232510189X

**Published:** 2025-12-29

**Authors:** Daniel Da Silva Pereira Curado, Amanda Oliveira Lyrio, Mariá Gonçalves Pereira Da Silva, Ana Carolina de Freitas Lopes, Lais Lessa Neiva Pantuzza, Everton Nunes da Silva

## Abstract

**Introduction:**

Since 2012, all drugs dispensed in the Brazilian Unified Health System (SUS) must be incorporated by the National Committee for Health Technology Incorporation (CONITEC). However, the characteristics of budget impact analyses (BIA) of incorporated drugs for chronic degenerative and rare diseases are unclear. Thus, our aim was to identify the profile of BIA of these drugs in the SUS between 2012 and 2024.

**Methods:**

Based on the 2012 to 2024 public reports of the CONITEC, data were collected on proposals for the incorporation of drugs for chronic degenerative and rare diseases into the SUS. The proposals were categorized by the type of incorporation proponent (public or private) and disease (rare or other chronic), by the presence of BIA, and by adoption of measured demand and active comparator. In addition, the median values of the eligible population and market share (first and fifth years) were calculated.

**Results:**

Among the 153 incorporated drugs, 53.6 percent (n=82) were proposed by private institutions, 36.6 percent (n=56) were for rare diseases, and 69.3 percent (n=106) presented a budget impact analysis. Among all BIA, 68.7 percent (n=79) adopted measured demand, frequently in other chronic diseases (76.1% [n=51] versus 58.3% [n=28]), and 25.0% (n=29) had no active comparator, more often in rare diseases (41.7% [n=20] versus 13.2% [n=9]). The median of the estimated population was higher for the other chronic diseases (27,237 versus 1,550). The median market share values were higher for rare diseases in both the first and fifth years (41.3% and 70.0% versus 20.0% and 50.0%).

**Conclusions:**

Most of the proposals presented BIA. The BIA for rare diseases used measured demand less frequently, had fewer active comparators, and smaller population medians, which may justify the higher market share rates. Since drugs for chronic degenerative and rare diseases are often high cost, knowing the profile of BIA is essential to improve CONITEC’s decision-making and to ensure the sustainability of the SUS.

